# Genotype-Phenotype Predictive Gap in Pre-Emptive Screening of DPD Deficiency: Improving Sensitivity

**DOI:** 10.3390/ph19101551

**Published:** 2026-09-30

**Authors:** Gerardo Salerno, Ottavia De Luca, Caterina Ilari, Maurizio Simmaco, Marina Borro

**Affiliations:** 1 Department of Neurosciences, Mental Health and Sensory Organs (NESMOS), Faculty of Medicine and Psychology, Sapienza University of Rome, 00189 Rome, Italy; gerardo.salerno@uniroma1.it (G.S.); maurizio.simmaco@uniroma1.it (M.S.); 2 Laboratory of Clinical Biochemistry and Molecular Biology, Sant’Andrea Hospital of Rome, 00189 Rome, Italy; o.deluca@ospedalesantandrea.it (O.D.L.); c.ilari@ospedalesantandrea.it (C.I.)

**Keywords:** fluoropyrimidines, dihydropyrimidine-dehydrogenase, DPD deficiency, *DPYD*, pharmacogenomics, 5-fluorouracil degradation assay

## Abstract

**Background/Objectives**: toxicity related to treatment with the anti-cancer drugs fluoropyrimidines (FP) is mainly driven by a deficiency in the dihydropyrimidine-dehydrogenase (DPD) enzyme activity. Standardized pharmacogenomic markers in the *DPYD* gene allow pre-emptive prediction and therapy adjustment in patients testing positive, but the rarity of these markers lowers screening sensitivity, leaving a significant part of at-risk individuals undetected. This study aimed to explore the diagnostic performance of genetic screening tests modelled to include unclassified rare *DPYD* variants and the common SNPs rs1801160 and rs2297595. **Methods**: this is a retrospective genotype-phenotype association study including 687 Italian subjects who underwent DPD-phenotyping by the 5-fluorouracil degradation assay (5-FUDR) and *DPYD* genotyping by full exon sequencing. According to their DPD-phenotype, subjects were grouped into a DPD deficiency group (DD, N = 94; >30% reduction in 5-FUDR) and a normal DPD activity group (NDA, N = 593). The phenotype was used as the reference standard to assess genotype-based functional prediction, and discordant genotype-phenotype cases were scored as false negatives or false positives. The phenotype (DD or NDA) prediction power of a genetic, binary (presence/absence) screening test including standardized pharmacogenomic markers (Test 1) was compared to binary tests including missense, nonsense, frameshift or splice-site rare variants (Test 2) or the co-occurrence of rs1801160 and rs2297595 (Test 3). Statistical significance was set at *p* < 0.05, correcting for multiple testing when appropriate. **Results**: positivity for all binary tests was significantly associated with the DPD deficiency phenotype (Test1: OR = 6; Test 2: OR = 4.85; Test3: OR = 4.9). Notably, overlap among subjects testing positive in the different tests was minimal. Combining all tests, a 3-fold increase in screening sensitivity was achieved compared to the reference Test 1 (from 12.77% to 35.11%), with a slight decrease in specificity from 97.64% to 91.74%. **Conclusions**: the proposed approach can refine the genetic predictivity of the DPD-deficiency phenotype in pre-emptive screening, capturing a significantly larger pool of biochemically at-risk patients.

## 1. Introduction

Solid tumours, most notably colorectal, gastric, pancreatic, breast, and head and neck cancers, are widely treated with fluoropyrimidines (FPs), including 5-fluorouracil (5-FU) and its pro-drugs capecitabine and tegafur [1,2]. Despite their anti-cancer efficacy, FP-related toxicities and life-threatening adverse drug reactions remain a major clinical concern. The discovery of an enzyme deficiency underlying the onset of these toxicities opened the way to pre-treatment screening to individualize therapy regimens in at-risk patients. Indeed, dihydropyrimidine dehydrogenase (DPD, encoded by the *DPYD* gene) catalyzes the rate-limiting step of 5-FU catabolism, inactivating about 80% of the administered dose. Reduced DPD activity impairs 5-FU elimination, exposing the patient to overdosage through an increased conversion into cytotoxic metabolites (e.g., FdUMP, FUTP) that inhibit thymidylate synthase and are incorporated into RNA/DNA [3]. This anti-metabolite pathway is not tumour-selective, affecting rapidly dividing normal tissues, such as the gastrointestinal mucosa and bone marrow, driving toxicity [4,5].

Testing for DPD deficiency has evolved as a screening method to identify patients likely to develop toxicity. The development of phenotyping assays, measuring directly or indirectly the actual enzyme activity, paralleled the implementation of genotyping assays, which predict deficiency based on *DPYD* markers. This is particularly relevant in colorectal cancer, where fluoropyrimidine-based regimens (e.g., FOLFOX, FOLFIRI, capecitabine monotherapy) constitute the backbone of both adjuvant and metastatic treatment and represent one of the most common indications for pre-emptive testing in clinical practice, although the same rationale extends to the other FP-treated tumour types mentioned above.

Whereas phenotyping captures the net effect of all genetic and non-genetic determinants of enzyme functionality, genotyping is an indirect, inferential method that predicts activity based on the functional annotation of a finite set of known variants. As a consequence, phenotyping can detect DPD deficiency even in the absence of a currently recognized pathogenic variant, while genotyping cannot capture non-genetic sources of variability but offers the advantage of being more standardized and scalable. Indeed, although several phenotypic assays have been developed, successfully reducing the rate and severity of FP toxicities, their clinical implementation remains limited, likely due to high equipment costs [6]. In our institution, a phenotyping assay using peripheral blood mononuclear cells, namely the 5-fluorouracil degradation rate (5-FUDR) [7], has been used for many years, in parallel with *DPYD* genotyping. The DPD deficiency detected by the 5-FUDR assay has been consistently associated with fluoropyrimidine toxicity, and the association between decreased 5-FUDR values and the presence of pharmacogenomic *DPYD* markers has been demonstrated as well [8,9,10,11].

In contrast to phenotyping, *DPYD* genotyping has rapidly spread worldwide, facilitated by the widespread availability of genotyping platforms. The genetic basis of DPD deficiency was identified early on [5,12,13], with several mutations in the *DPYD* gene accounting for the onset of FPs toxicity. Currently, four variants are cleared as recommended pre-emptive tests by the main drug regulatory agencies [14,15]: the splice-site variant rs3918290, the missense variants rs67376798 and rs55886062 and the HapB3 haplotype (composed of rs75017182 and rs56038477, which are in nearly complete linkage disequilibrium) (Table 1). The main pharmacogenomic authorities, i.e., the Clinical Pharmacogenetics Implementation Consortium (CPIC) and the Dutch Pharmacogenetics Working Group (DPWG) have provided an extended list (Table 1) of *DPYD* variants classified as having reduced or null function and have established a standardized framework to convert genotypes into a clinically actionable phenotype, based on computing an activity score (AS). Normal function alleles have an AS = 1, reduced function alleles have an AS = 0.5 and null function alleles have an AS = 0) [16,17,18]. Specifically, CPIC recommends avoiding fluoropyrimidines in patients with AS = 0 (or using a markedly reduced dose with early therapeutic drug monitoring if no alternative is available), reducing the starting dose by 50% in patients with AS = 0.5, and reducing it by 25–50% in patients with AS = 0.75, with dose titration guided by toxicity in all cases [19].

Despite the recognized effectiveness of genotyping standardized variants, their sensitivity is limited by their low minor allele frequency (MAF). Consequently, FP toxicity still develops in approximately 10–20% of marker-negative subjects [20]. This is partly explained by the occurrence of rare non-classified or population-specific disruptive mutations, as increasingly reported by full-gene or exome *DPYD* sequencing [21,22]. Clinically, this genotype-phenotype discrepancy is highly relevant: patients who test negative on standard pre-emptive *DPYD* panels but are phenotypically DPD-deficient remain undetected and are consequently exposed to exceeding fluoropyrimidine plasma concentration, placing them at risk of toxicity.

A further part of the discrepancy between the observed phenotype and the genotype-based prediction may arise from unresolved interaction or cumulative effects among common single nucleotide polymorphisms (SNPs) in the *DPYD* gene. Among common SNPs, the rs1801160 (c.2194G>A; p.V732I) and the rs2297595 (c.496A>G; p.M166V), are classified as normal function alleles by the CPIC/DPWG system. Even though both have been repeatedly associated with a mild decrease in DPD activity and with fluoropyrimidine toxicity, the slightness of the effect and the heterogeneity of reported outcomes justify the CPIC classification [23,24,25]. In a previous study, we reported a significant association between a haplotype structure involving the aforementioned SNPs and a consistently decreased 5-FUDR [9].

With the aim to increase the fraction of pre-emptively identified DPD deficiencies, this retrospective study on an Italian cohort (N = 687) assessed the association between DPD deficiency and (i) the presence of a CPIC-classified variant, as a reference benchmark; (ii) the presence of non-classified, rare missense, nonsense, frameshift or splice-site variants detected by *DPYD* exon sequencing; and (iii) the co-occurrence of the common SNPs rs1801160 and rs2297595. To achieve this, the study cohort was stratified into a DPD deficiency group (DD, N = 94) and a normal DPD activity group (NDA) defined by the 5-FUDR metric. We then evaluated the genotype-phenotype prediction power of different modelled genetic screening tests: (i) Test 1: Presence/absence of a CPIC-classified variant; (ii) Test 2: presence/absence of a non-classified, rare (MAF < 0.01) missense, nonsense, frameshift or splice-site variant; and (iii) Test 3: presence/absence of both rs1801160 and rs2297595. The genotype-phenotype prediction power of extended screening panels obtained by merging Test 1–3 was also assessed.

## 2. Results

### 2.1. Demographics, Phenotype and Genotype Distribution

Following identification and removal of duplicate samples, the study cohort included 687 Italian subjects (45.71% female). The distribution of both age and 5-FUDR values was non-normal, and thus non-parametric statistics were applied. The median age was 68 years (range 20–90, IQR:14) and median 5-FUDR was 1.45 ng/mL/10^6^ cells/min (the unit of measurement will be abbreviated as “U” hereafter), with a range of 0–2.24 (IQR: 0.55). Using a cut-off value of 5-FUDR < 1.015 U (corresponding to a reduction in DPD activity >30%) subjects were assigned to either the DPD deficiency group (DD, N = 94) or the normal DPD activity group (NDA, N = 593). No statistically significant differences in age or gender distribution between DD and NDA groups were observed.

Genotype frequencies of the identified CPIC-classified variants and of the common SNPs rs1801160 and rs2297595 are reported in Table 2. No deviation from Hardy–Weinberg equilibrium was detected for any marker. Non-parametric analysis by the Mann–Whitney U or Kruskal–Wallis tests showed significant differences in the distribution of the 5-FUDR values among genotypes for all variants except for the rs56038477 and the rs67376798. The observed effect size was moderate-to-large for rs67376798 and small-to-moderate for rs56038477 but imprecisely estimated given the small number of variant carriers, consistent with the corresponding wide confidence interval.

The phenotype-predictive power of a binary genetic test modelled by assuming the presence/absence of a CPIC-classified variant as a positive testing criterion for DPD deficiency (Test 1) is shown in Table 3. A significant association of a positive test with the DD phenotype was observed, giving an OR = 6.0 (95% CI: 2.7–13.5) for the presence of a DPD deficiency.

### 2.2. Association Between DPD Deficiency and Non-Classified, Rare Variants

The heterozygous presence of missense (N = 26), frameshift (N = 1) or splice-site rare variants (N = 1) were detected in a total of 32 subjects, 13 of whom (40.6%) were classified as DD (Table 4). Among these, only one patient also carried a CPIC variant, namely the HapB3. Few variants were identified in more than one subject: they are indicated in Table 4, where 5-FUDR values measured in each carrier are reported.

Only four mutations were annotated in the ClinVar database [26] as pathogenic/likely pathogenic, three of which were identified in the DD group. Of these, the p.P86L was present in two subjects who showed an approximately 50% decrease in 5-FUDR compared to the median value of the overall population (0.68 U and 0.73 U vs. 1.45 U), whereas the p.A551T was present in a patient with complete DPD deficiency (5-FUDR = 0). This represented a unique occurrence of total enzymatic deficiency among more than 2000 patients phenotyped at our institution over several years.

Among the variants annotated as “uncertain significance”, the p.P519S was detected in two patients with about half the median 5-FUDR (0.72 U and 0.77 U, respectively).

The phenotype-predictive power of the binary genetic test modelled by assuming the presence/absence of a non-classified rare variant as a positive testing criterion for DPD deficiency (Test 2) is shown in Table 3. Fisher’s exact test highlighted a significantly increased proportion of DD subjects in the sub-group of patients testing positive, giving an OR = 4.85 (95% CI: 2.3–10.2) for the presence of a DPD deficiency. The significance level was similar after removing one patient who also bore the HapB3 variant (*p* = 3.33 × 10^−4^; OR = 4.42, 95% CI: 2.07–9.44).

### 2.3. Association Between DPD Deficiency and Co-Occurrence of rs1801160 and rs2297595

As expected, single-SNP analysis confirmed a statistically significant but moderate effect of the two common variants and DPD deficiency (Table 2).

The potential cumulative effect of the co-occurrence of the two polymorphisms on DPD deficiency was assessed by applying a dominant genetic model to dichotomize the study cohort according to the unphased genotype combination at the rs1801160 and rs2297595 loci. All subjects carrying at least one variant allele at both loci where coded as “positive”, while the remaining combinations were coded as “negative”, yielding a total of 29 positive (all being double heterozygous) and 658 negative subjects. As shown in Table 3 (Test 3), the proportion of DD subjects was significantly enriched in the positive-test group (OR = 4.9; 95% CI: 2.3–10.7). Of note, only 4 out of 29 positive patients carried a CPIC-classified variant (N = 1) or an additional rare variant (N = 3).

The statistical significance was maintained even after removing from the total sample 57 subjects carrying a CPIC-classified variant or an additional rare variant (*p* = 7.4 × 10^−4^; OR = 5.0, 95% CI: 2.1–11.8). A significant difference between positive and negative subjects was also detected for the distribution of the crude 5-FUDR values using the Mann–Whitney U-test (*p* = 3.32 ×10^−8^; median 5-FUDR 1.05 U vs. 1.46 U, respectively).

Having established a significant association between co-occurrence of rs1801160 and rs2297595 and the DD phenotype (Test 3, Table 3), we used logistic regression to formally test whether double carriers additional risk beyond the combination of the two markers’ individual effects, and whether each marker remained independently associated with the DD phenotype after adjustment for demographic factors and for the other genetic markers.

A multivariable model including age, sex, CPIC-classified variant status (Test 1), rare variant status (Test 2), rs1801160, rs2297595 and their interaction term showed that age (OR = 1, 95% CI: 0.97–1.02, *p* = 0.79) and sex (OR = 0.93; 95% CI: 0.58–1.48; *p* = 0.75) were not significantly associated with the DD phenotype, whereas the presence of a CPIC-classified variant (OR = 8.18; 95% CI: 3.48–19.21; *p* < 0.0001), a rare variant (OR = 4.63; 95% CI: 2.10–10.23; *p* = 0.0001), rs1801160 (OR = 2.09; 95% CI: 1.11–3.95; *p* = 0.023) and rs2297595 (OR = 3.15; 95% CI: 1.77–5.61; *p* = 0.0001) remained independently associated with DD after mutual adjustment; the interaction term remained non-significant (OR = 1.14; 95% CI: 0.38–3.43; *p* = 0.81). Ethnicity/ancestry was not included as a covariate, as the cohort was drawn from a single-centre Italian population with no recorded ethnic heterogeneity.

### 2.4. Genotype-Phenotype Prediction Power of Differently Modelled Genetic Screening Test

The negative and positive predictive values (NPV, PPV), sensitivity and specificity of the three differently modelled genetic screening panels are reported in Table 3.

The screening panel testing for the presence/absence of a CPIC-classified variants (Test 1) yielded the highest specificity but a low sensitivity, as expected.

The screening panels testing for the presence/absence of rare (MAF < 0.01 in the Non-Finnish European population) variants (Test 2) and the presence/absence of the co-occurrence of rs1801160 and rs2297595 (Test 3), evaluated independently, exhibited a similar overall diagnostic performance.

The extended Tests 4 and 5, created by merging the positivity criteria from Tests 1–2 or from Tests 1–3, respectively, showed a sharp increase in sensitivity (nearly 2-fold and 3-fold increases, respectively), with a shallower decrease in specificity, which remained above 90%. Positivity for both Test 4 and 5 was significantly associated with an increased risk of the DD phenotype (OR = 5.8; 95% CI: 3.2–10.4; and OR = 6.0 95% CI: 3.6–10.0, respectively).

## 3. Discussion

Pharmacogenomic testing has changed the approach to FP treatment: prospective genotype-guided dose individualization has been shown to reduce the relative risk of severe (grade ≥ 3) toxicity by approximately 36–54% in *DPYD* variant carriers compared with historical, non-genotyped controls [27]. Nevertheless, a major proportion of adverse reactions remains unpredictable when screening exclusively for the four core *DPYD* variants (Table 1). Despite their high specificity, their clinical sensitivity is inadequate to fully rule out the onset of severe, life-threatening toxicities. This diagnostic limitation is fundamentally driven by their low population frequency. Besides the CPIC-classified variants, which have been clearly assigned to a functional status (normal, reduced or null activity) (Table 1), several additional mutations are now being identified through the increasing use of next-generation sequencing for *DPYD* exon or full-gene sequencing [21,22]. Unfortunately, the rarity of these variants impairs our ability to definitively classify them into discrete functional categories due to inadequate statistical power.

This study addresses the phenotypic (DPD deficiency), rather than clinical (toxicity), component of this predictive gap; as such, it represents a preliminary step toward clinical risk assessment, which will require validation against direct fluoropyrimidine toxicity outcomes. Our approach deliberately avoids stratifying rare variants based on current, often evolving database annotations, which are frequently biassed by low statistical power and poor correlation with the biochemical phenotype. Instead, we propose a model based on an a priori protein-disruptive potential, evaluating all missense, nonsense, frameshift or splice-site, rare mutations as a single pooled layer, since these specific variant categories carry the highest biological probability of deleterious functional effects. Under this light, the presence of an unclassified rare variant should not be interpreted as an indication for drug dosage reduction, but strictly as a clinical warning that warrants closer monitoring—for instance, prompting confirmatory phenotypic testing or heightened vigilance for early signs of toxicity during the first treatment cycle—rather than an empirical dose modification.

We detected a non-classified, rare mutation in approximately 4.6% (32/687) of subjects, and only four out of 28 variants had a pathogenic/likely pathogenic annotation on a main clinical variation database such as ClinVar. In silico functional prediction for these variants using online tools such as CADD values or Predict-SNP [28,29] did not correctly identify a significant proportion of DD (DPD deficiency) cases as defined by the phenotypic assay 5-FUDR. Furthermore, inspection of Table 4 suggests additional criticisms for the standardization process of *DPYD* genotype-phenotype correlations. First, most carriers (19/32, 59.4%) of a *DPYD* mutation have normal enzyme activity; furthermore, some mutations are present in both DD and NDA patients, confirming that unresolved genetic and non-genetic mechanisms contribute to determining the biochemical phenotype, as further highlighted by the case of a patient bearing the mutation p.Ala551Thr in heterozygosity with a complete loss of enzyme activity.

Taken together, these findings underscore the diagnostic challenge of *DPYD* rare variants: most remain functionally uncharacterized and are therefore reported, if at all, as VUS (variants of uncertain clinical significance), a category whose presence alone cannot be reliably translated into a predicted phenotype. Despite this uncertainty, in the analyzed cohort, the absolute frequency of rare variants with higher protein-disruptive potential, such as missense, nonsense, frameshift and splice-site variants, is significantly increased in DD patients, suggesting that their identification may serve as a preliminary alert to keep clinical focus high. This hypothesis needs clinical validation.

The second attempt in this study, which aimed to expand the fraction of genotype-predicted DPD deficiencies, also arose from considering the unresolved genetic complexity underlying the biochemical phenotype. Generally, single-SNP analyses may yield conflicting or statistically non-significant results when an unresolved haplotype structure serves as the primary driver of the phenotype. In practice, standard clinical genotyping routinely yields unphased data, leaving the exact haplotype structure (*cis* vs. *trans* configuration) undetermined. Rather than overlapping with traditional haplotype studies, our work intentionally shifts the focus toward evaluating the clinical effect of unphased genotype combinations. In the case of the DPD phenotype, it should be borne in mind that the active enzyme is a homodimer [30], and therefore the co-occurrence of SNPs in both *cis* and *trans* configurations may exhibit differential functional effects compared to single-SNP effects. Thus, we explored the possibility of an independent effect of the simultaneous presence of at least one mutant allele in both the rs1801160 and rs2297595 loci, mimicking the real-world condition of a diagnostic test reporting a list of unphased genotypes for multiple loci. Our results confirmed an increased functional effect of such co-occurrence compared to single-SNP effects, with potential clinical significance, as suggested by the diagnostic performance of the positive test (Table 3, Test 3). It should be noted that the MAFs reported for rs1801160 and rs2297595 vary across different ethnicities (0.017 to 0.12 and 0.017 to 0.17, respectively) [25]. In our study, the observed MAFs were around 10% for both SNPs, (similar to those previously reported in an Italian cohort) [6], increasing the number of co-carrying subjects and allowing the detection of an association with the DPD-deficiency phenotype. To formally test whether this co-occurrence effect reflected a true statistical interaction rather than the independent, multiplicative combination of the two variants’ individual effects, we fitted a logistic regression model including both SNPs and an interaction term (dominant coding, as in Test 3). The interaction term was not statistically significant, indicating that the increased risk observed in double carriers is compatible with a multiplicative combination of two independent risk alleles rather than evidence of biological synergy.

Of note, patients testing positive for Test 1 (CPIC-classified variant) showed negligible overlap with those testing positive for Test 2 (non-classified, rare variant) or Test 3 (co-occurrence of rs1801160 and rs2297595). The mutual independence was further confirmed in a multivariable logistic regression model adjusting simultaneously for age, sex, and all genetic markers, in which each marker remained independently and significantly associated with the DD phenotype, while age and sex were not significant covariates.

Given this independence, merging the three tests into combined screening panels (Test 4: Test 1 + Test 2; Test 5: Test 1 + Test 2 + Test 3) substantially increased the rate of DPD deficiency detection, with the most inclusive panel (Test 5) improving sensitivity from 12.77% to 35.11% while maintaining acceptable specificity (91.74%) (Table 3).

These data suggest that the diagnostic gap between the genotype-predicted and actual phenotypes can be partially bridged by accounting for both unclassified rare variants and the combined effect of common single nucleotide polymorphisms.

We are aware of the limitations of this study, particularly the complete lack of direct clinical toxicity data, which prevented us from performing population stratification according to clinical toxicity grading. Consequently, our findings describe the genetic predictivity toward the biochemical phenotype (DPD deficiency) rather than direct clinical adverse events, and we cannot conclude that the expanded genetic panel proposed here improves the prediction of fluoropyrimidine toxicity. Decreased DPD activity, as measured by 5-FUDR, has nonetheless been consistently associated with fluoropyrimidine toxicity risk in independent studies [8,9,10,11], supporting its relevance as a biochemical surrogate, even though direct toxicity validation in the present cohort was not possible.

Considering the rarity of the genetic markers of DPD deficiency, we propose a complementary approach for pre-emptive screening, transforming heterogeneous data into a binary test, namely the presence/absence of one among several single markers. This approach allows us to increase the sub-groups’ numerosity and perform adequate statistical analysis; on the other hand, CPIC-classified markers with different activity scores are collapsed into a single layer. We want to remark that this was a methodological choice strictly aimed at evaluating overall diagnostic performance allowing direct comparison with the additional tests proposed, and that the model does not imply a clinical pooling of individual markers. The same caveat applies to the pooled rare-variant test (Test 2, see also Section 3), which is intended as an exploratory screening layer rather than a substitute for individual clinical interpretation of each variant.

This binary test model is easily reproducible and thus could be rapidly applied to re-evaluate available clinical datasets, possibly including toxicity reports, to validate the present findings.

Recent literature highlights a growing concern that standard *DPYD* screening panels are predominantly based on European genetic variants [31,32,33]. However, evaluating and standardizing functional impact of *DPYD* mutations is difficult across any ethnicity due to their rarity. Our proposed approach addresses this limitation by deliberately avoiding reliance on population-specific allele frequency or classification databases: rare missense, nonsense, frameshift or splice-site variants are pooled and evaluated as a single risk category based on their predicted disruptive location within the gene, rather than on their annotation in any given reference population; this approach can therefore in principle be applied across genetically diverse cohorts. Conversely, the impact of the co-occurrence of the common SNPs rs1801160 and rs2297595 (Test 3) is expected to be more sensitive to population-specific allele frequencies: since both are common variants whose MAFs vary widely across ethnicities (0.017–0.12 and 0.017–0.17, respectively) [34], populations with lower MAFs will have fewer co-carriers, reducing the statistical power to detect an association with the DPD-deficient phenotype, whereas populations with higher MAFs—such as the Italian cohort studied here (~10% for both SNPs)—are more likely to show a detectable effect. Population-specific linkage disequilibrium patterns may further influence the cis/trans configuration of co-occurring alleles, potentially modulating the net functional effect given the homodimeric structure of the DPD enzyme. Thus, diagnostic performance for the prediction of the DPD phenotype remains to be validated in populations of different ancestries.

## 4. Materials and Methods

### 4.1. Data Collection

Data were collected retrospectively, enrolling Italian patients (N = 687) who underwent a DPD phenotyping assay, namely the 5-FUDR, and a *DPYD* genotyping assay by exon sequencing, between April 2022 and March 2026. An oncologic diagnosis was implicit in the test request, but no additional clinical data (tumour type, staging, comorbidities, treatment, outcome) were available.

The study was conducted in accordance with the Declaration of Helsinki and was approved by the local ethical committee (Comitato Etico Territoriale Lazio Area 1, Prot. 0030/2025). Written informed consent was obtained from the participants.

The 5-FUDR values and the *DPYD* variant lists identified in each participant were extracted from the Laboratory Information System (LIS). Analytical validation parameters of the 5-FUDR assay (PBMC isolation, storage conditions, intra-/inter-assay variability, quality-control criteria, and lower limit of quantification) are reported in the referenced methodological publication [7].

The *DPYD* genotyping test was performed by sequencing all *DPYD* exons and exon-intron boundaries, using the Thermo Fisher Scientific (Waltham, MA, USA) Ion GeneStudio S5 System, a next-generation sequencing (NGS) platform. Variant calling was executed by the Ion Reporter Software, version 5.22 (Thermo Fisher Scientific). The analytical panel therefore allowed the identification of any variant in the coding region, including the CPIC-classified variants reported in Table 1. The test result consistently included, as standard outputs, the genotypes for the following variants: rs3918290, rs56038477 (tagging the HapB3 marker), rs67376798, rs55886062, rs1801160, and rs2297595. An “additional variant” field was filled in the case of a rare missense, nonsense, frameshift or splice-site variant was identified. Rare variants were defined as those with a MAF < 0.01 for the Non-Finnish European population in the gnomAD database, a subpopulation matching the ancestry of our Italian cohort [34]. Splice-site variants were defined as variants occurring within a range of ± 5 base pairs from the exon-intron junction. For descriptive purposes, rare variants were additionally cross-referenced against the ClinVar database [26], which classifies genetic variants into five tiers—pathogenic, likely pathogenic, uncertain significance (VUS), likely benign, and benign—generally following ACMG/AMP interpretation criteria [35].

No clinical data or demographic characteristics other than age, gender, and ethnicity were available.

### 4.2. Statistics

Statistical analysis was performed using SPSS software version 27 (IBM Statistics, Armonk, NY, USA). Hardy–Weinberg equilibrium was evaluated using the online tool SNPStats [36].

The distribution of age and 5-FUDR values was assessed graphically and through the Shapiro–Wilk test, which confirmed a statistically significant deviation from normality for both parameters. The association between continuous non-parametric variables (age and 5-FUDR) was assessed by Spearman correlation analysis. The association between continuous and categorical variables was assessed by the Mann–Whitney U-test or the Kruskal–Wallis test, as appropriate. Effect sizes (Cohen’s d for two-genotype comparisons; Cohen’s f for three-genotype comparisons) with 95% CI (normal-approximation CI for d; percentile bootstrap CI, 3000 resamples, for f, derived from bootstrapped η^2^) are reported in Table 2 alongside *p*-values.

To evaluate the genotype-phenotype association, the study cohort was dichotomized into a DPD deficiency group (DD, N = 94) and a normal DPD activity group (NDA, N = 593). Specifically, a reduction in the median 5-FUDR > 30% was chosen (yielding the cut-off value of <1.015 U relative to the cohort median of 1.45 U). This strategy aligns with the CPIC pharmacogenomic guidelines, which set an Activity Score (AS) of 1.5—corresponding to a 75% residual enzyme activity—as the clinical threshold for initial drug dosage reduction [14,15]. Furthermore, the threshold is consistent with independent phenotyping studies: a >30% reduction from the population mean (i.e., activity below 70% of the mean) has similarly been used to define significant DPD deficiency in lymphocyte-based biochemical assays [37,38].

The phenotype-predictive power of genetic screening tests was evaluated using three differently modelled binary tests (presence/absence), as follows. Test 1: Presence of a CPIC-classified variant reported in Table 1 (positive/negative); Test 2: presence of at least one rare missense, nonsense, frameshift or splice-site variant; Test 3: co-occurrence of at least one mutant allele at both the rs1801160 and rs2297595 loci. Sensitivity, specificity, positive predictive value (PPV), and negative predictive value (NPV) were computed for: each test, independently; the pooled tests 1 and 2 (named “Test 4”); and the pooled tests 1, 2 and 3 (named “Test 5”). The predictive performance of the five screening strategies for the DPD deficiency phenotype was evaluated on 2 × 2 contingency tables, using odds ratios with Woolf 95% confidence intervals and two-sided Fisher’s exact tests; PPV, NPV, sensitivity and specificity were derived from the same tables.

To formally test whether the co-occurrence effect observed for Test 3 reflected a statistical interaction beyond the independent, multiplicative contribution of the two variants, a multivariable logistic regression model, including age, sex, CPIC-classified variant status (Test 1), rare variant status (Test 2), rs1801160, rs2297595 and the rs1801160 × rs2297595 interaction term, was fitted to assess whether each marker was independently associated with the DD phenotype after mutual adjustment.

The two-sided statistical significance was set at a *p*-value < 0.05. Bonferroni correction for multiple testing was applied when appropriate.

## 5. Conclusions

Prospective studies incorporating direct clinical toxicity outcomes, and validation in independent and more genetically diverse cohorts, will be needed to determine whether improved detection of the DPD-deficiency phenotype translates into reduced fluoropyrimidine toxicity, and to define its optimal place alongside phenotypic testing in routine pre-treatment screening. More broadly, our findings reinforce the notion that ethnic and population-specific variability—observed here for *DPYD* and documented for other pharmacogenomic markers—should be explicitly factored into the design of pre-emptive screening panels, favouring population-tailored, cost-effective testing strategies over a uniform, one-size-fits-all approach.

## Figures and Tables

**Table 1:** List of CPIC-classified variants.

	rsID	Aliases	AS
Tier 1-(Minimum set)	rs3918290 *	c.1905+1G>A; IVS 14+1G>A; * 2A	0
	rs55886062 *	c.1679T>G; p.I560S; * 13	0
	rs75017182 rs56038477	(HapB3 *) c.1129-5923C>G c.1236G>A	0.5/0.75 ^†^
	rs67376798 *	c.2846A>T; p.D949V	0.5
	rs115232898	c.557A>G, p.Y186C	0.5
	rs146356975	c.868 A>G; p.K290E	0.5
	rs112766203	c.2279 C>T; p.T760I	0.5
Tier 2-(Optional)	rs72549309	c.295_298delTCAT; p.F100fs; * 7	0
	rs1801266	c.703 C>T; p.R235W; * 8	0
	rs186169810	c.1314 T>G; p.F438L	0.5
	rs72549304	c.1475 C>T; p.S492L	0
	rs59086055	c.1774 C>T; p.R592W	0
	rs55674432	c.2639 G>T; p.G880V	0

AS: Activity Score. * Recommended by EMA/FDA as fluoropyrimidine pre-treatment screening; ^†^ a recent online update (July 2026) on https://www.clinpgx.org/guideline/PA166251462 (accessed on 25 August 2026) suggests modifying the AS from 0.5 to 0.75.

**Table 2:** Genotype frequencies of the CPIC-classified variants and the common SNPs rs1801160 and rs2297595 in the study cohort (N = 687).

*rsID* (Aliases)	Genotype Frequency	Median 5-FUDR (IQR)	*p* *	HW	Effect Size (95% CI)
rs3918290 (c.1905+1G>A; IVS 14+1G>A; *2A)	GG 681 GA 6	1.45 (0.55) 0.87 (0.21)	**8.60 × 10^−4^**	1.00	d = −1.72 (−2.53 to −0.91)
rs56038477 (c.1129-5923C>G; Hap B3)	CC 676 CT 11	1.45 (0.56) 1.16 (0.49)	0.50	1.00	d = −0.47 (−1.07 to 0.12)
rs67376798 (c.2846A>T; p.D949V)	TT 679 TA 8	1.45 (0.56) 1.11 (0.65)	0.19	1.00	d = −0.79 (−1.49 to −0.09)
rs115232898 (c.557A>G; p.Y186C)	AA 686 AG 1	1.45 (0.55) 0.64 (-)	na	1.00	na
rs1801160 (c.2194G>A; p.V732I)	CC 553 CT 128 TT 6	1.55 (0.54) 1.24 (0.52) 1.11 (0.49)	**2.28 × 10^−7^**	0.83	f = 0.221 (0.147–0.296)
rs2297595 (c.496A>G; p.M166V)	TT 541 TC 142 CC 4	1.49 (0.52) 1.29 (0.60) 1.03 (0.46)	**7.50 × 10^−5^**	0.12	f = 0.175 (0.090–0.257)

* Bonferroni-corrected *p*-value from a two-sided Mann–Whitney U test (2-genotype markers) or Kruskal–Wallis test (3-genotype markers) on 5-FUDR values across genotype groups. Significant *p*-values are shown in bold. Effect sizes are reported as Cohen’s d (two-genotype comparisons) or Cohen’s f (three-genotype comparisons).

**Table 3:** Distribution of NDA and DD subjects by test positivity/negativity and diagnostic performance for each genetic test.

		NDA (N)	DD (N)	*p* *	OR ^†^ (CI95%)	PPV (CI95%)	NPV (CI95%)	% Sen. (CI95%)	%Sp. (CI95%)
Test 1 (CPIC-classified variants)	Pos Neg	14 579	12 82	**2.0 × 10^−4^**	6.00 (2.7–13.5)	46.15 (28.76–64.54)	87.59 (84.86–89.89)	12.77 (7.46–21.00)	97.64 (96.08–98.59)
Test 2 (unclassified, rare variants)	Pos Neg	19 574	13 81	**5.0 × 10^−4^**	4.85 (2.3–10.2)	40.63 (25.52–57.74)	87.63 (84.89–89.94)	13.83 (8.26–22.24)	96.80 (95.05–97.94)
Test 3 (co-occurrence of rs1801160 and rs2297595)	Pos Neg	17 576	12 82	**7.75 × 10^−4^**	4.90 (2.3–10.7)	41.38 (25.51–59.26)	87.54 (84.79–89.85)	12.77 (7.46–21.00)	97.13 (95.46–98.20)
Test 4 (Test 1 + Test 2)	Pos Neg	33 560	24 70	**1.14 × 10^−7^**	5.80 (3.2–10.4)	42.11 (30.19–55.02)	88.89 (86.20–91.11)	25.53 (17.80–35.19)	94.44 (92.29–96.01)
Test 5 (Test 1 + Test 2 + Test 3)	Pos Neg	49 544	33 61	**4.10 × 10^−10^**	6.00 (3.6–10.0)	40.24 (30.30–51.06)	89.92 (87.26–92.07)	35.11 (26.22–45.17)	91.74 (89.24–93.69)

* Bonferroni-corrected *p*-values from Fisher’s exact test; ^†^ Mantel-Haenszel common OR. Significant *p*-values are shown in bold.

**Table 4:** List of missense, nonsense, frameshift, and splice-site rare variants identified in the study cohort. The 5-FUDR value observed in each carrying subject is reported.

Locus (GRCh37)	dbSNP	Coding	Consequence	5-FUDR (U)	ClinVar
chr1:98348894	rs766635900	c.76G>A	p.A26T	0.91	na
chr1:98348884	rs528768620	c.86G>A	p.R29H	1.13	VUS
chr1:98206016	rs757342874	c.253G>T	p.A85S	1.46	na
chr1:98206012	rs568132506	c.257C>T	p.P86L	0.68; 0.73	P/LP
chr1:98164976	rs2524392204	c.611C>T	p.S204F *	1.89	na
chr1:98164969	no rs	c.617_618insT	p.L206Ffs * 8	1.19	na
chr1:98144726	rs45589337	c.775A>G	p.K259E ^†^	0.86; 1.54	VUS/B/LB
chr1:98060698	rs748266854	c.875C>T	p.A292V	1.10; 1.61; 1.72	na
chr1:98058945	rs577302209	c.959-2A>G	Splice site	0.98	LP
chr1:98058830	rs779557503	c.1072C>T	p.R358C	1.59	LP
chr1:98058829	rs573299212	c.1073G>A	p.R358H ^†^	1.54	na
chr1:98058785	rs772906420	c.1117G>A	p.V373I	1.24	na
chr1:98039437	rs61622928	c.1218G>A	p.M406I	1.38; 1.38	VUS/B
chr1:97981467	rs672601282	c.1555C>T	p.P519S	0.72; 0.77	VUS
chr1:97981407	rs142619737	c.1615G>A	p.G539R	0.86; 1.22	VUS/LB
chr1:97981371	rs777425216	c.1651G>A	p.A551T	0	LP/VUS
chr1:97771751	rs145548112	c.2161G>A	p.A721T	1.03	VUS
chr1:97770928	rs146529561	c.2186C>T	p.A729V	0.89	VUS
chr1:97770895	rs1667093380	c.2219G>A	p.G740D	0.82	na
chr1:97700497	rs1411946304	c.2353C>A	p.L785M	1.93	na
chr1:97700472	rs547099198	c.2378C>T	p.T793I	2.04	VUS
chr1:97658663	rs753419296	c.2584G>A	p.G862R *	1.89	na
chr1:97564155	rs147545709	c.2656C>T	p.R886C	0.81	na
chr1:97564097	rs1650432721	c.2714C>T	p.S905L	1.21	na
chr1:97547971	rs748639205	c.2822T>G	p.V941G ^¥^	1.38	VUS
chr1:97544582	rs760485592	c.3028C>T	p.P1010S ^¥^	1.38	na
chr1:97544563	rs773868825	c.3047G>A	p.G1016D	1.14	na
chr1:97544549	rs148799944	c.3061G>C	p.V1021L	0.98	na

*, ^†^, ^¥^ Identical symbols indicate variants detected in the same individual (compound heterozygote): * p.S204F and p.G862R; ^†^ p.L259E and p.R358H; ^¥^ p.V941G and p.P1010S. U: unit measure, corresponding to ng/mL/10^6^ cells/min; VUS: variant of uncertain significance; B: Benign; LB: Likely benign; P: Pathogenic LP: Likely pathogenic; na: no available annotation.

## Data Availability

The minimum dataset supporting the findings of this study has been deposited in the Zenodo repository, https://doi.org/10.5281/zenodo.23013957. Data are available upon request, for non-profit and academic research purposes.

## Abbreviations

The following abbreviations are used in this manuscript:

CI	Confidence interval
CPIC	Clinical Pharmacogenetics Implementation Consortium
DD	DPD deficiency
DPD	Dihydropyrimidine-dehydrogenase
DPWG	Dutch Pharmacogenetics Working Group
EMA	European Medicines Agency
FDA	US Food and Drug Administration
FPs	Fluoropyrimidines
5-FUDR	5-fluorouracil degradation rate
IQR	Interquartile range
MAF	Minor allele frequency
NDA	Normal DPD activity
NPV	Negative predictive value
OR	Odds ratio
PPV	Positive predictive value
SNP	Single nucleotide polymorphism
VUS	Variant of uncertain significance

## References

[1] Sonbol M.B., Bekaii-Saab T., Larsen C.M. (2025). Fluoropyrimidine Therapy in Gastrointestinal Cancer: Balancing Survival Benefits and Cardiotoxicity Risks. JACC Cardio Oncol..

[2] Deac A.L., Burz C.C., Bocsan I.C., Buzoianu A.D. (2020). Fluoropyrimidine-induced cardiotoxicity. World J. Clin. Oncol..

[3] Longley D.B., Harkin D.P., Johnston P.G. (2003). 5-fluorouracil: Mechanisms of action and clinical strategies. Nat. Rev. Cancer.

[4] Barin-Le Guellec C., Lafay-Chebassier C., Ingrand I., Tournamille J.-F., Boudet A., Lanoue M.-C., Defossez G., Ingrand P., Perault-Pochat M.-C., Etienne-Grimaldi M.-C. (2020). Toxicities associated with chemotherapy regimens containing a fluoropyrimidine: A real-life evaluation in France. Eur. J. Cancer.

[5] Ezzeldin H., Diasio R. (2004). Dihydropyrimidine dehydrogenase deficiency, a pharmacogenetic syndrome associated with potentially life-threatening toxicity following 5-fluorouracil administration. Clin. Colorectal Cancer.

[6] Deac A.L., Burz C.C., Bocşe H.F., Bocşan I.C., Buzoianu A.D. (2020). A review on the importance of genotyping and phenotyping in fluoropyrimidine treatment. Med. Pharm. Rep..

[7] Lostia A.M., Lionetto L., Ialongo C., Gentile G., Viterbo A., Malaguti P., Paris I., Marchetti L., Marchetti P., De Blasi A. (2009). A liquid chromatography-tandem mass spectrometry method for the determination of 5-Fluorouracil degradation rate by intact peripheral blood mononuclear cells. Ther. Drug Monit..

[8] Mazzuca F., Borro M., Botticelli A., Mazzotti E., Marchetti L., Gentile G., La Torre M., Lionetto L., Simmaco M., Marchetti P. (2016). Pre-treatment evaluation of 5-fluorouracil degradation rate: Association of poor and ultra-rapid metabolism with severe toxicity in a colorectal cancer patients cohort. Oncotarget.

[9] Gentile G., Botticelli A., Lionetto L., Mazzuca F., Simmaco M., Marchetti P., Borro M. (2016). Genotype-phenotype correlations in 5-fluorouracil metabolism: A candidate *DPYD* haplotype to improve toxicity prediction. Pharmacogenomics J..

[10] Onesti C.E., Botticelli A., La Torre M., Borro M., Gentile G., Romiti A., Lionetto L., Petremolo A., Occhipinti M., Roberto M. (2017). 5-Fluorouracil degradation rate could predict toxicity in stages II-III colorectal cancer patients undergoing adjuvant FOLFOX. Anti-Cancer Drugs.

[11] Botticelli A., Onesti C.E., Strigari L., Occhipinti M., Di Pietro F.R., Cerbelli B., Petremolo A., Anselmi E., Macrini S., Roberto M. (2017). A nomogram to predict 5-fluorouracil toxicity: When pharmacogenomics meets the patient. Anti-Cancer Drugs.

[12] Tuchman M., Stoeckeler J.S., Kiang D.T., O’Dea R.F., Ramnaraine M.L., Mirkin B.L. (1985). 419 Familial Pyrimidinemia and Pyrimidinuria, A New Pharmacogenetic Disorder?. Pediatr. Res..

[13] Diasio R.B., Beavers T.L., Carpenter J.T. (1988). Familial deficiency of dihydropyrimidine dehydrogenase. Biochemical basis for familial pyrimidinemia and severe 5-fluorouracil-induced toxicity. J. Clin. Investig..

[14] European Medicines Agency (EMA) (2020). Direct Healthcare Professional Communication (DHPC): 5-Fluorouracil (i.v.), Capecitabine and Tegafur Containing Products: Pre-Treatment Testing to Identify DPD-Deficient Patients at Increased Risk of Severe Toxicity. https://www.ema.europa.eu/en/medicines/dhpc/5-fluorouracil-iv-capecitabine-tegafur-containing-products-pre-treatment-testing-identify-dpd#documents-sectio.

[15] US Food and Drug Administration (FDA) https://www.fda.gov/drugs/resources-information-approved-drugs/safety-announcement-fda-highlights-importance-dpd-deficiency-discussions-patients-prior-capecitabine.

[16] Caudle K.E., Thorn C.F., Klein T.E., Swen J.J., McLeod H.L., Diasio R.B., Schwab M. (2013). Clinical Pharmacogenetics Implementation Consortium guidelines for dihydropyrimidine dehydrogenase genotype and fluoropyrimidine dosing. Clin. Pharmacol. Ther..

[17] Lunenburg C.A.T.C., van der Wouden C.H., Nijenhuis M., Rhenen M.H.C.-V., de Boer-Veger N.J., Buunk A.M., Houwink E.J.F., Mulder H., Rongen G.A., van Schaik R.H.N. (2020). Dutch Pharmacogenetics Working Group (DPWG) guideline for the gene-drug interaction of DPYD and fluoropyrimidines. Eur. J. Hum. Genet..

[18] Pratt V.M., Cavallari L.H., Fulmer M.L., Gaedigk A., Hachad H., Ji Y., Kalman L.V., Ly R.C., Moyer A.M., Scott S.A. (2024). DPYD Genotyping Recommendations: A Joint Consensus Recommendation of the Association for Molecular Pathology, American College of Medical Genetics and Genomics, Clinical Pharmacogenetics Implementation Consortium, College of American Pathologists, Dutch Pharmacogenetics Working Group of the Royal Dutch Pharmacists Association, European Society for Pharmacogenomics and Personalized Therapy, Pharmacogenomics Knowledgebase, and Pharmacogene Variation Consortium. J. Mol. Diagn..

[19] Amstutz U., Henricks L.M., Offer S.M., Barbarino J., Schellens J.H., Swen J.J., Klein T.E., McLeod H.L., Caudle K.E., Diasio R.B. (2018). Clinical Pharmacogenetics Implementation Consortium (CPIC) Guideline for Dihydropyrimidine Dehydrogenase Genotype and Fluoropyrimidine Dosing: 2017 Update. Clin. Pharmacol. Ther..

[20] Ho T.T., Smith D.M., Aquilante C.L., Cicali E.J., El Rouby N., Hertz D.L., Imanirad I., Patel J.N., Scott S.A., Swain S.M. (2025). A Guide for Implementing DPYD Genotyping for Systemic Fluoropyrimidines into Clinical Practice. Clin. Pharmacol. Ther..

[21] Moyer A.M., Lundquist E., Thoreson E.K., Kluge M.L., Kotzer K.E., Love E.M., Glessner H.L., Arneson S.K., Langman L.J., Baudhuin L.M. (2026). DPYD Sequencing Identifies More Clinically Relevant Variants as Compared to Targeted Genotyping. Clin. Chem..

[22] De Luca O., Salerno G., De Bernardini D., Torre M.S., Simmaco M., Lionetto L., Gentile G., Borro M. (2022). Predicting Dihydropyrimidine Dehydrogenase Deficiency and Related 5-Fluorouracil Toxicity: Opportunities and Challenges of *DPYD* Exon Sequencing and the Role of Phenotyping Assays. Int. J. Mol. Sci..

[23] Otero-Torres S., Rodríguez-Mauriz R., Fort-Casamartina E., Clopés-Estela A., Soler-Rotllant F., Fontanals-Martínez S., Montero-Pérez O. (2025). *DPYD* Genotyping, Fluoropyrimidine Dosage and Toxicity: An Umbrella Review of Systematic Reviews. Pharmaceuticals.

[24] Božina N., Bilić I., Ganoci L., Šimičević L., Pleština S., Lešnjaković L., Trkulja V. (2022). DPYD polymorphisms c.496A>G, c.2194G>A and c.85T>C and risk of severe adverse drug reactions in patients treated with fluoropyrimidine-based protocols. Br. J. Clin. Pharmacol..

[25] Hamzic S., Schärer D., Offer S.M., Meulendijks D., Nakas C., Diasio R.B., Fontana S., Wehrli M., Schürch S., Amstutz U. (2021). Haplotype structure defines effects of common DPYD variants c.85T > C (rs1801265) and c.496A > G (rs2297595) on dihydropyrimidine dehydrogenase activity: Implication for 5-fluorouracil toxicity. Br. J. Clin. Pharmacol..

[26] ClinVar. https://www.ncbi.nlm.nih.gov/clinvar/search/?gene=dpyd&assembly=GRCh38.

[27] Henricks L.M., Lunenburg C.A.T.C., de Man F., Meulendijks D., Frederix G.W.J., Kienhuis E., Creemers G.-J., Baars A., Dezentjé V.O., Imholz A.L.T. (2018). DPYD genotype-guided dose individualisation of fluoropyrimidine therapy in patients with cancer: A prospective safety analysis. Lancet Oncol..

[28] CADD—Combined Annotation Dependent Depletion. https://cadd.gs.washington.edu/snv.

[29] Bendl J., Musil M., Štourač J., Zendulka J., Damborský J., Brezovský J. (2016). PredictSNP2: A Unified Platform for Accurately Evaluating SNP Effects by Exploiting the Different Characteristics of Variants in Distinct Genomic Regions. PLoS Comput. Biol..

[30] Lohkamp B., Voevodskaya N., Lindqvist Y., Dobritzsch D. (2010). Insights into the mechanism of dihydropyrimidine dehydrogenase from site-directed mutagenesis targeting the active site loop and redox cofactor coordination. Biochim. Biophys. Acta.

[31] Gil-Rodriguez A., Recarey-Rama S., Rodríguez-Viyuela A., Cruz R., Barros F., Carracedo A., Maroñas O. (2025). Differences in DPYD Population Frequencies Observed in Galicians Compared to Europeans and Spanish from PhotoDPYD Study. Pharmaceuticals.

[32] White C., Scott R.J., Paul C., Ziolkowski A., Mossman D., Ackland S. (2021). Ethnic Diversity of DPD Activity and the *DPYD* Gene: Review of the Literature. Pharmgenomics Pers. Med..

[33] Chan T.H., Zhang J.E., Pirmohamed M. (2024). *DPYD* genetic polymorphisms in non-European patients with severe fluoropyrimidine-related toxicity: A systematic review. Br. J. Cancer.

[34] gnomAD The Genome Aggregation Database. https://gnomad.broadinstitute.org.

[35] Richards S., Aziz N., Bale S., Bick D., Das S., Gastier-Foster J., Grody W.W., Hegde M., Lyon E., Spector E. (2015). Standards and guidelines for the interpretation of sequence variants: A joint consensus recommendation of the American College of Medical Genetics and Genomics and the Association for Molecular Pathology. Genet. Med..

[36] Solé X., Guinó E., Valls J., Iniesta R., Moreno V. (2006). SNPStats: A web tool for the analysis of association studies. Bioinformatics.

[37] Milano G., Etienne M.C., Pierrefite V., Barberi-Heyob M., Deporte-Fety R., Renée N. (1999). Dihydropyrimidine dehydrogenase deficiency and fluorouracil-related toxicity. Br. J. Cancer.

[38] Etienne M.C., Lagrange J.L., Dassonville O., Fleming R., Thyss A., Renée N., Schneider M., Demard F., Milano G. (1994). Population study of dihydropyrimidine dehydrogenase in cancer patients. J. Clin. Oncol..

